# Co-designing a cardiac rehabilitation program with knowledge users for patients with cardiovascular disease from a remote area

**DOI:** 10.1186/s12913-024-11321-4

**Published:** 2024-07-31

**Authors:** Jessica Bernier, Mylaine Breton, Marie-Eve Poitras

**Affiliations:** 1 Department of Family Medicine and Emergency Medicine, University of Sherbrooke, Saguenay-Lac-St-Jean, 305, Saint-Vallier, Chicoutimi, Québec G7H 5H6 Canada; 2 CRMUS Research Chair On Optimal Professional Practices in Primary Care, Centre Intégré Universitaire de Santé et de Services sociaux du, Saguenay–Lac-St-Jean, Saguenay, Canada; 3 Department of Community Health Sciences, Université de Sherbrooke, Sherbrooke, QC Canada; 4 Canada Research Chair in Clinical Governance on Primary Health Care, Université de Sherbrooke, 150 Pl. Charles-Le Moyne, Longueuil, QC J4K 0A8 Canada

**Keywords:** Cardiac rehabilitation program, Cardiovascular disease, User-centered design, Remote areas

## Abstract

**Background:**

Cardiovascular disease is the leading cause of death worldwide. Cardiac rehabilitation (CR) programs are recognized as effective in reducing the burden of cardiovascular disease. However, CR programs are offered inequitably across regions and are available in less than 15% of remote areas worldwide. The main goal of this study was to design a CR program adapted to the contexts of remote areas to improve the service offered to patients.

**Methods:**

We used an iterative user-centered design approach to understand the user context and services offered in cardiac rehabilitation in remote areas. We conducted two co-design processes with knowledge users in two remote regions. Two advisory committees were created in each of these regions, comprising managers (*n* = 6), healthcare professionals (*n* = 12) and patients (*n* = 2). We utilized the BACPR guidelines and the Hautes Autorités de santé operational model to support data collection in coding sessions to develop the CR program. We conducted four cycles of co-design with each of the committees to develop the cardiac rehabilitation program. Qualitative data were analyzed iteratively after each cycle.

**Results:**

The co-design process resulted in developing a prototype cardiac rehabilitation program similar in both regions. It is based on a contextualized six-phase pathway of care designed for remote regions. For each phase 0 to 6 of the care pathway, knowledge users were asked to describe how to offer these phases in remote areas. Participants made structural changes to phases 0, 2, 3 and 4 in order to overcome staffing shortages in remote areas. These changes make it possible to decentralize cardiac rehabilitation expertise away from specialized centers, to ensure equity of service across the territory. Therapeutic patient education was integrated into phase 4 to meet patients' needs. Participants suggested that three follow-up offerings could come from nursing services to increase access to the cardiac rehabilitation program (primary care, home care, special chronic disease programs) in patients' home communities.

**Conclusion:**

The co-design process enables us to meet the needs of remote regions in program development. This final program can be the subject of future implementation research.

## Introduction

Cardiovascular disease (CVD) is the leading cause of death worldwide [1]. Among these, ischemic heart disease is the leading cause of death and morbidity [2]. Following ischemic heart disease, it is recommended to adhere to a cardiac rehabilitation (CR) program, which is a range of services aimed at reducing the burden associated with CVD [3]. CR programs are necessary to "*influence favourably the underlying cause of cardiovascular disease, as well as to provide the best possible physical, mental, and social conditions, so that the patients may, by their efforts, preserve or resume optimal functioning in their community and through improved health behaviour, slow or reverse progression of the disease.* (p. 1)" (British Association for Cardiovascular Prevention and Rehabilitation, [BACPR] [4]).

Turk-Adaiwai et al. [5] identified the range of CR programs available worldwide through a cross-sectional study conducted in 203 countries. CR programs are available in 54% of the countries surveyed, are predominantly offered in urban areas and close to hospitals (71.6%) offering cardiac procedures required for CVD treatment. According to these authors, 19 million RC program service points are still missing [5]. Only 12% of remote areas offered CR programs [5]. The authors highlight the presence of an inequitable and insufficient CR offer between countries that need to meet patients' needs. This gap is particularly marked in remote regions. Recent studies have shown that the absence of a CR program in remote areas is detrimental to people with CVD [6]. These people are left, among other things, with unmet needs after a cardiac event. These needs may be related to psychosocial health, health education, lifestyle, or medical risk management [6].

The main factors identified as hindering the deployment of a CR program may be related to financial and human resources, as well as difficulties in referring patients [5]. Difficulties in referring patients to the CR program can be explained, among other things, by the longer delays in managing patients living in remote areas compared with patients living close to tertiary centers [5, 7, 8]. Some studies also report a failure to transmit patient-related information between the tertiary and primary centers, which impairs patient referral and delays patient management after a cardiac event [9, 10]. Another recent study points out that the referral process within a patient's trajectory to CR programs needs to be better-defined, and eligibility guidelines are often absent [9]. Some studies point out that living far from a hospital center that offers a CR program is a barrier to patient enrollment due to the distance involved in participating in said programs [5, 11].

Access to interdisciplinary teams is also a major issue [5]. In remote areas, interdisciplinary teams are often limited in human resources compared with teams in specialized centers [5, 12]. A CR program offering is considered optimal when provided by an interdisciplinary team [12] composed of, among others, kinesiologists, nutritionists, nurses and psychologists [4]. Healthcare professionals deliver CR programs that can be offered through complementary service delivery modalities, enabling greater access [11]. These different service delivery modalities can be provided face-to-face in a CR center, at home and via telephone follow-ups, but also in synchronous telehealth mode with professionals, in asynchronous mode with support through virtual applications or a patient record portal managed by healthcare professionals [11]. Implementing CR programs with different service delivery modalities is essential for better patient outcomes [13].

Although the literature abounds in describing the various components of a CR program, there remains a lack of consensus on the set of components that must be present for said CR program to be optimal for patients. However, there is a convergence of a CR program based on three essential phases [14–16]. Phase 1 focuses on early mobilization in the hospital setting and preventing immobility during hospitalization. Phase 2 represents access to various services focused on CR programs offered by an interdisciplinary team. Phase 3 is maintaining lifestyle changes over the long term, supported by sporadic use of community services [14–16]. Although three phases may be present, CR programs focus more on phase 2 by offering different activities to improve the health of the person with CVD [17, 18]. Phase 2 focuses on risk factor management, health education, medication, physical activity and smoking cessation [4].

The British Association for Cardiovascular Prevention & Rehabilitation (BACPR) [4, 19] suggests six care pathway CR program phases. The three phases mentioned above are present in the six BACPR model phases. Phase 0 enables patient identification for hospitalization and early referral to CR. Phase 1 manages patient referral and early recruitment to CR. Phases 2 and 3 facilitate patient assessment and the development of a service plan for CR. Phase 4 is the provision of CR by an interdisciplinary team. Phase 5 is the final post-CR assessment of the patient, and Phase 6 is the discharge from the CR program and transition to the community. The BACPR [4] vision is much more comprehensive, following the patient from hospitalization to completion of the CR program, with no loss of patient follow-up. These phases ensure adherence and uptake of a CR program [20]. Throughout these six phases, it is recommended that components related to behaviour management and health education, psychosocial health management, medical risk management, and lifestyle risk management be included (BACPR, 4). In 2023, BACPR [21] also modernized its terminology by offering complementary service delivery modalities, 1) face-to-face, 2) distance learning and 3) hybrid, to broaden the CR offer, which was centralized in specialized centers offering CR programs.

The scientific literature and clinical recommendations from CR frameworks, including that of the BACPR, provide little guidance on how a CR program can be implemented and operationalized in a remote context [4, 9, 11]. However, a few models for co-designing a CR program have been reported in the literature (hybrid CR program via telehealth; a web platform to support health education) [22, 23]. Little information is available on the elements that have been contextualied for remote areas, apert form support for resumption of physical activity via a synchronous or asynchronous mode, remote support, reduced monitoring time by professionals and multimodal provision (e.g. home, center-based, telehealth, in-person) [22, 23].

Our literature review revealed no established patient referral pathways for enrolling patients in a cardiac rehabilitation program in remote areas. Yet, CR programs must be supported by guidelines and a clearly established trajectory of care and services, including an early referral process from the patient's hospitalization to CR program healthcare professionals [9]. To this end, Beleigoli et al. [24] have developed a CVD patient referral to the CR program for people living more than 50 KM away, considering their preference for follow-up delivery modalities (e.g. telephone, face-to-face and virtual). However, they do not describe the components offered by the CR program in the specific context of the follow-up modalities chosen by patients. The gaps identified in the literature are the absence of an RC program in remote areas and the need for a care and service pathway within an RC program.

The services offered through a cardiac rehabilitation (CR) program for people living in remote areas are often not tailored to their local context and sometimes even absent. This limits patient participation in a CR program and ultimately reduces its effectiveness for patients. Thus, the overall goal of this study was to design a CR program adapted to the contexts of remote areas to improve the service offered to patients. The study was conducted at two sites simultaneously to provide context for mapping available services to CVD patients after PCI in two remote regions.

The specific objectives of this study were:

1. To understand the context of knowledge users by describing services offered to CVD patients in remote areas.
2. To develop prototypes of a CR program adapted to the context of remote areas.
3. Present the development process of a prototype CR program co-constructed in partnership with knowledge users in a region far from tertiary centers.

## Methods

### Design

We used user-centered design (UCD) to conceptualize a prototype of a CR program adapted to two regions far removed from tertiary centers in collaboration with knowledge users. UCD is an iterative process that actively seeks and incorporates user feedback [25]. It involves four stages: understanding the user context, specifying user requirements, designing solutions, and evaluating the design against the requirements [25].

### Conceptual model

**Fig. 1:**
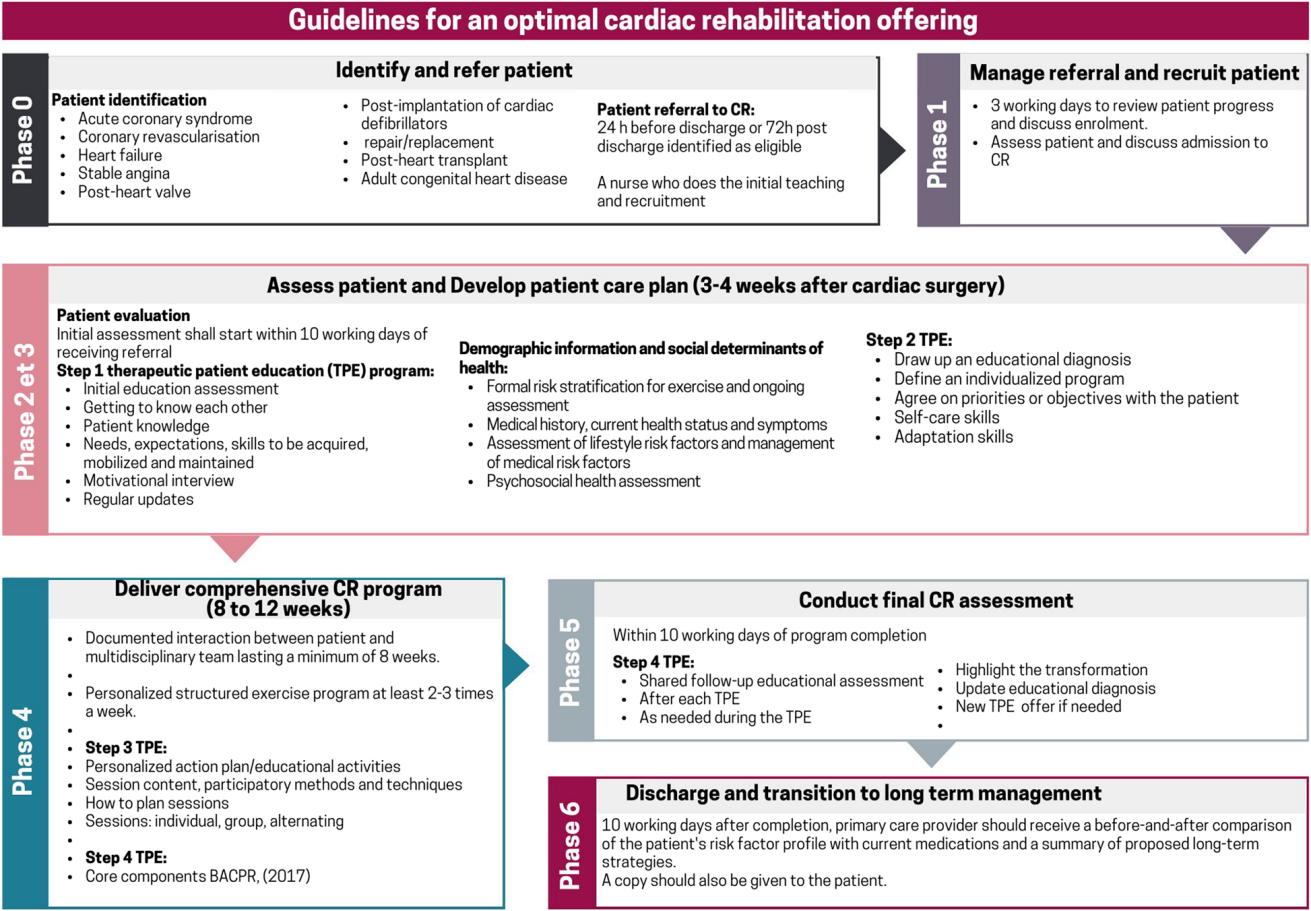
Diagram of guidelines derived from CR operational models adapted RC (BACPR, [4], HAS, [26])

The development of a CR program was based on the six phases of a pathway of care, as well as the four essential educational components of the BACPR model [4] activity management. However, the essential components of the BACPR model [4] are too underdeveloped to operationalize the delivery of these essential components in the CR program. Thus, the research team also relied on the methodological guide of the Hautes Autorités de santé [26] with the concept of therapeutic patient education (TPE) found in a CR program and each of the six phases. Fig. 1 shows the schematic diagram of a CR program and the educational elements that must be addressed within a CR program. These two models complement each other to provide an overall view of a CR offering that is not explicitly described in the literature. This schematization was used to guide the co-creation of the CR program presented in this study.

### Study context

We carried out this project in two regions of Quebec (Canada) located more than two hours away from a specialized tertiary cardiology center where percutaneous coronary interventions (PCI) occur. Site 1 has a population of almost 282,000, covers an area of nearly 96,000 km^2^ and includes five main municipalities [27]. Site 2 has a population of over 90,000, covers an area of over 230,000 km^2^ and comprises five main municipalities [27]. In both regions, some services are available for CVD patients. However, many services recommended in a CR care pathway are unavailable.

### Participants and recruitment

We used the convenience sampling strategy to recruit knowledge users to reflect the reality of the program to be developed [28]. Three types of knowledge users were recruited: healthcare professionals (e.g. nutritionists, nurses, kinesiologists), managers and patients with CVD. The research team collaborated with managers to recruit healthcare professionals. The healthcare professionals supported the research team with patient recruitment. Inclusion criteria were to be healthcare professionals, have expertise with CVD patients, and have held a permanent position in primary care for at least six months. Patients must have had an MI within the last two years before the study started, and managers must have held their positions for at least six months.

### Data collection

**Fig. 2:**
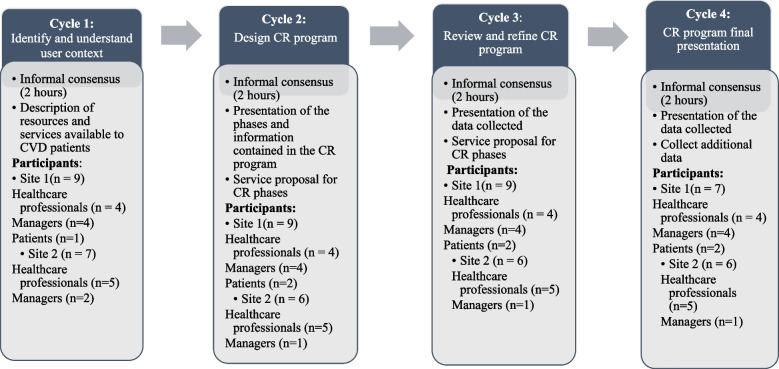
The 4-cycle co-design process to develop the prototype CR program with participants

We met with participants over four meeting cycles to design a prototype CR program. Fig. 2 illustrates the data collection process for each co-design cycle in developing the prototype CR program with knowledge users. This figure also illustrates the data collection method, the themes discussed, and the participants in each cycle.

We used the informal consensus method for the four iterative co-design cycles [29]. Informal consensus is an unstructured deliberative method without a rating or voting system. It allows committee members to express their opinions, and special attention is paid to patients. We collected data from January to May 2023, with the number of participants varying from cycle to cycle according to their clinical occupation and topic discussed. This aligns with the UCD co-design model [30].

Cycle 1 allowed us to understand the context of our participants and, thus, the services available to CVD patients. The services offered were placed within the phases of a CR program to better contextualize the services for participants with CVD.

Data collection in Cycles 2 and 3 was based on the elements outlined in Fig. 2 of the CR program guidelines diagram. During discussions, we used this figure to mobilize exchanges around how they could offer the CR program, as suggested by BACPR and Hautes Autorités en Santé, in their area. We documented knowledge users requirements during the development of the CR program prototype. Cycle 4 was used to present the CR program's final prototype and gather final comments for improvement. All meetings were recorded via the TEAM platform.

### Data capture and analysis

We used the session analysis method based on notes and recordings [31]. We utilized a Word table to record textual notes during iterative cycles. After each cycle, the recordings were transcribed, and the data was included in a Word table. Additionally, the recordings were used to synthesize the data. Following Cycle 1, we mapped the services offered in CR in the two regions. After Cycle 2, the first author used the field notes and recordings to create the initial version of the CR program prototype in collaboration with the two other authors. This led to Cycle 3, during which the prototype of the CR program was finalized. For Cycle 3, we re-analyzed the field data and recorded data to enhance the prototype. In Cycle 4, we presented the final prototype program for feedback. Participant feedback was incorporated to modify and improve the prototype CR program during the iterative co-design process.

## Results

Four cycles of co-design led to the development of a prototype for a remote CR program. Table 1 presents the socio-demographic characteristics of participants for both sites. Unfortunately, despite our recruitment efforts, no patients participated in the discussion cycles for site 2.

**Table 1:** Sociodemographic characteristics of the study sample (*n* = 20)

Professionals’ characteristics (*n* = 18)	*N* = 11 (%) Site 1	*N* = 7 (%) Site 2
Sex		
Men	1 (9,09)	1 (14,2)
Women	10 (90,9)	6 (85,7)
Educational level		
Bachelor’s degree	8 (72,7)	5 (71,4)
Master’s degree	3 (27,3)	2 (28,5)
Experiential knowledge		
Registered nurse	3 (27,3)	3 (42,8)
Nurse Practitioner	1 (9,09)	1 (14,2)
Nutritionist	1 (9,09)	1 (14,2)
Kinesiologist	1 (9,03)	0
Health Services Manager	5 (45,5)	0
Decision maker	0	2 (28,5)
Years of experience as professional		
16 years and more	5 (45,4)	4 (57,1)
15 years and less	6 (54,5)	3 (42,8)
Year of experience in the practice setting		
16 years and more	3 (27,2)	2 (28,5)
15 years and less	8 (72,7,)	5 (71,4)
Patients ‘characteristics (*n* = 2)	*N* = 2 (%)	*N* = 0
Patients		0
Men	2 (100)	
Number of Myocardial Infarction		
1	1(50)	
2	1(50)	
Educational level		
College	1(50)	
University	1(50)	
Occupation		
Full-time work	2(100)	
Other chronic health problems		
2 or less	1(50)	
3 or more	1(50)	
Physical activity		
3 times/week and less	1(50)	
3 times/week and more	1(50)	

### Objective 1: Understand the context of knowledge users by describing services offered to CVD patients in remote areas

Discussions during Cycle 1 allowed us to understand the services offered to CVD patients at each site. Both sites offer services corresponding to phases 2, 3 and 4 of the optimal care pathway, as suggested by the BACPR. Table 2 summarizes the services available to CVD patients in each study region.

**Table 2:** Description of current CR services based on the CR pathway of care phases

	**Phase 0** Identify and refer patient	**Phase 1** Manage referral and recruit patient	**Phase 2** Assess patient **Phase 3** Develop patient care plan	**Phase 4** Deliver comprehensive CR programme	**Phase 5** Conduct final CR assessment	**Phase 6** Discharge and transition to long term management
Site 1	Patient information guides Video capsules	No service	Cardiac risk stratification with the cardiologist Encounter with kinesiologist	Physical activity program supervised 3 times a week by kinesiologist	No service	No service
Site 2	No service	No service	Cardiac risk stratification with the internal medicine specialist	Nutrition follow-up by nutritionist Blood pressure follow-up by nurse Family doctor follow-up 2 months after MI	No service	No service

The participants reported that these phases were not structured within a comprehensive CR program, i.e. within a pathway of care, nor supported by specific CR guidelines as described in Fig. 1. The diagnostic test of cardiac exercise risk stratification for the patient (phases 2 and 3) is offered for both sites, allowing assessment of cardiovascular health during physical exertion. After this risk stratification, the patient can be directed towards physical activity under the supervision of a kinesiologist, who will help the patient learn how to exercise after a cardiac event. This risk stratification was only performed at Site 1.

More specifically, for site 1, an information guide is given to the patient after the cardiac procedure in the tertiary center, along with an Internet link to view two information capsules at home (phase 0). However, the information guide was not deployed in all regional hospitals. Diagnostic cardiac risk stratification is performed one month after the percutaneous coronary intervention (PCI). At this point, patients meet with the kinesiologist (phases 2 and 3). The kinesiologist then recommends patients to a supervised physical activity program offered at two points of service for phase 4. However, an inequity of services is present within this region, as the kinesiology service depends on the patient's place of residence (Phase 4). Within the same region, patients living in a town where there are kinesiologists offering supervised physical activity will be able to participate, compared with patients living in a more distant town who will not have access if a kinesiologist is absent or does not offer this service, for example.

For site 2, diagnostic cardiac risk stratification is performed and planned one month after the PCI (phase 2). No referral to the kinesiology service is made due to lack of services. Sporadically, medical follow-up with the family physician and blood pressure monitoring for patients referred by the family physician are carried out (Phase 4). Nutritional follow-up is offered without systematic referral after MI. Nutritional follow-up varies from patient to patient and within the region. It is provided at certain points of service only.

### Objective 2: Develop a prototype of a CR program adapted to the context of remote regions

**Fig. 3:**
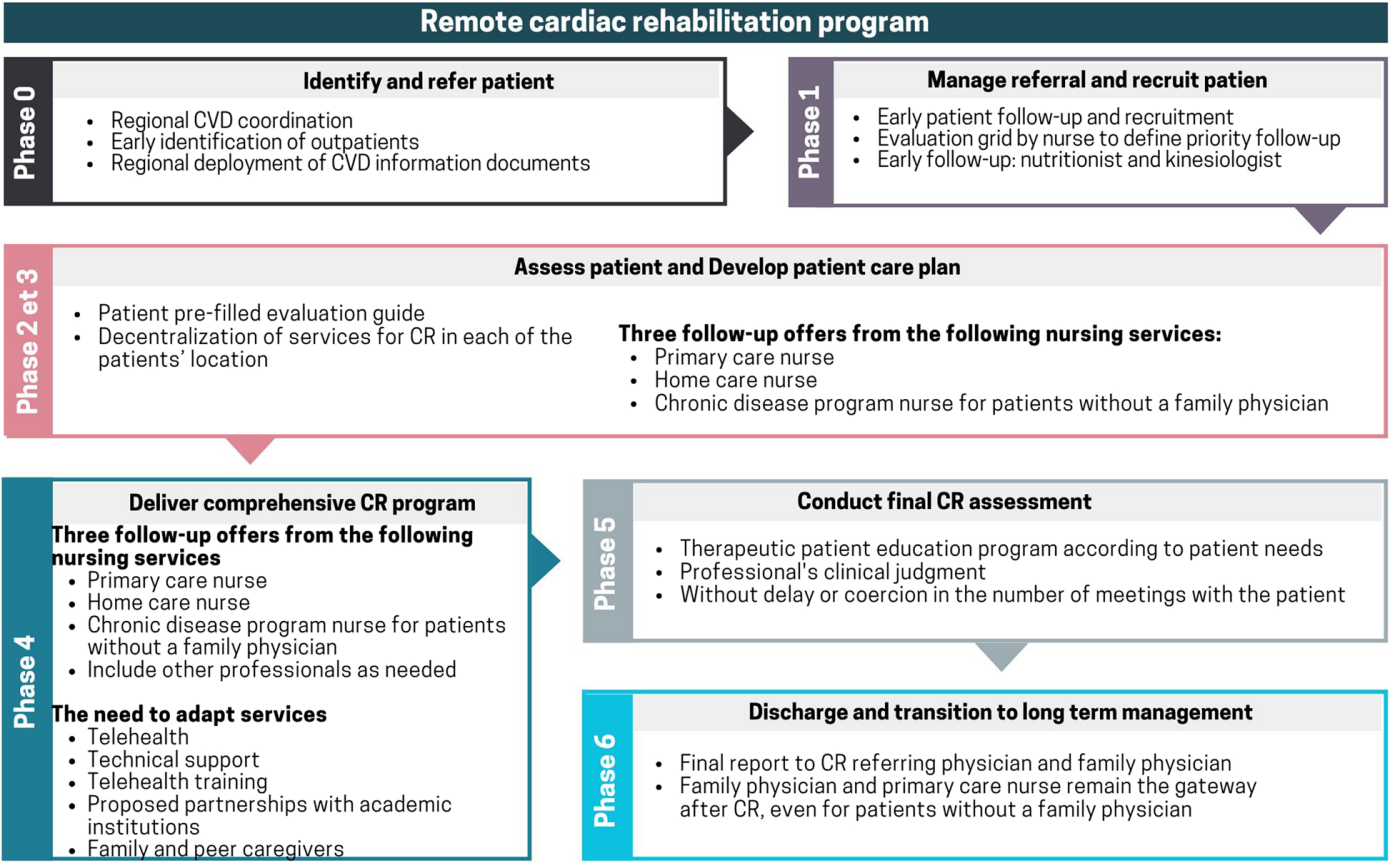
Schematic representation of the prototype CR program for a remote region

The results reported below are those generated by the two discussion cycles, Cycle 2 and Cycle 3. Although the CR program development process was carried out at two different sites, participants identified similar issues and needs in developing the CR program. As a result, it was possible to produce a single CR program map for both sites. The diagram of the CR program is shown in Fig. 3.

During the exchanges, several participants mentioned their fears that the development of the CR program would not be possible. Site 1 participants reported that the CR model developed by the BACPR (2017) is frightening for professionals working in remote regions due to material, human and financial resource issues: "*It's difficult for us; we have issues with professional resources, orphan patients without a family doctor and the vastness of the region*" [site 1]. Participants felt it would be impossible for them to offer CR services in the way the BACPR suggests. During the discussions, participants mentioned priority elements that could be implemented in their context, such as a regional nurse coordinator for patient referral, early follow-up with the nutritionist and kinesiologist, decentralization of tertiary services to patients' home communities, and three different nursing follow-up modalities.

There was also a discrepancy in perceptions of CVD patient services at Site 1 between the participants. On the one hand, tertiary center managers said they had good services and good tools: "(before the pandemic*) we were excellent in our services*" [manager, site 1]. On the other hand, healthcare professionals and patients reported not having a kinesiology service and information documents available at sites far from the tertiary center: "*No, we don't offer this service, we don't have a kinesiologist, and we don't have the teaching documents*"[ manager, site 1].

### Phase 0: Identify and refer patient

Participants mentioned the importance of having a single point of contact. They illustrated this by setting up a nurse as a regional CVD program coordinator. The nurse's role would be to 1) receive all patient referrals to the CR program following hospitalization for CVD and 2) identify patients early on for the CR program to better coordinate referrals, regardless of the point of entry. " *Patients navigate through different hospitals, so we need a single point of contact to receive all referrals*" [Decision maker site 1–2]. Nurse were agree with manager.

Site 1 participants mentioned the importance of disseminating information guides that inform patients about CVD and surgical intervention in each management trajectory for patients with chronic diseases (e.g. diabetes clinic) and in the various care facilities throughout the region covered by the CR service offering. "*We don't have information guides in our remote area* " [nurse, site 1].

### Phase 1: Manage referrals and recruit patients

Participants mentioned the importance of early follow-up after hospitalization by the nurse and CVD program coordinator. During this phase 1, the nurse manages the referrals received, and contacts patients to carry out an initial assessment to identify any possible health problems patients may have encountered since hospital discharge. At this point, the nurse carries out early recruitment of patients deemed eligible for the CR program. Patients at Site 1 mentioned that referral management and communication with the patient would provide a safety net after the PCI, should health issues arise. It would also allow healthcare professionals to respond to their early needs, such as the symptoms they were experiencing or their concerns about the follow-up appointment. "*It would be reassuring for us (patients) to have a follow-up with the nurse, to tell us the next steps and to talk if we have a health problem. We must go to the emergency room if we don't have a family physician*" [patient, site 1].

Healthcare professionals and managers at Site 1 reported the importance of implementing a patient priority care assessment grid, enabling them to evaluate problems since discharge and prioritize patients with urgent needs. " *We need an evaluation grid to know which patients should be prioritized for rapid follow-up*" [nurse, site 1]. Participants also stressed the importance of introducing early follow-up in nutrition, kinesiology and social work at all service points in each patient's locality. " *I don't have to wait until phase 4 to see patients[…] after they've stopped working, I don't see them anymore, they're working* "[nutritionist, site 1]. " *It would be reassuring to start the follow-up with the kinesiologist for our travels while waiting for the risk stratification*." [patient, site 1]. Participants also mentioned that after completing phase 1 and recruiting patients, the nurse coordinator would need to delocalize the patient's referral for CR follow-up with the local CR charge nurse near the patient's residence. " *Services need to be decentralized to ensure fairness throughout the region"* [nurse, site 1].

### Phases 2 and 3: Assess patient and develop patient care plan

For some patients, the distance for cardiac risk stratification and follow-up with the tertiary center nurse requires more than a two-hour drive. Participants mentioned the importance of being able to perform cardiac risk stratification in their community due to medical restrictions on driving after cardiac surgery (e.g. PCI and CABG). Risk stratification should be offered according to where the patient lives, either in a tertiary center or the hospital in the patient's home area. According to the participants, offering phase 2 and subsequent phases in each locality seems essential. *" I'm not allowed to drive, and I have to drive more than 2 h to stratify the risk "* [patients, site 1].

Participants report the importance of having CR services offered close to home by professionals other than those provided by the tertiary center. Tertiary center services are to be decentralized to each local chronic disease site in patients' home communities from phase 2 onwards. Participants reported the need for patients to complete a document in preparation for the meeting with the doctor and nurse. Data in the document includes, for example, data on medication side effects, weight, questions and, among other things, retrosternal pain. At the time of the encounter, this data from the patient could be used to pinpoint possible problems, as perceived by the patient, but also monitor blood pressure, pulse, weight, presence of pain, possible medication side-effects, psychological issues, and so on. *" Having a completed document would assist in the evaluation before surgery to identify any potential issues"* [nurse, site 1].

Participants suggested developing a follow-up offer close to home by nurses trained locally in CR who can practice in primary care or chronic disease clinics and outside tertiary CR centers. The opportunity to offer follow-up in the various delocalized sectors of tertiary CR centers in primary care through nurses from remote areas would enable more patients to be reached. According to the participants, nurses working in primary care medical clinics, in the chronic disease sector and in-home care could be mobilized to work with CVD patients. In addition to better meeting the needs of patients in remote areas, this would enable the expertise of nurses working in specialized tertiary CR centers to be decompartmentalized towards nurses working in primary care or the chronic disease sector. These follow-ups could be integrated into their role and activities as primary care and chronic disease nurses. *" We can handle cardiac rehabilitation for patients; we know their medical chart and available resources*" [nurse, site 1 and 2].

The three follow-up offers could come from the following nursing services: 1) CR follow-up by the nurse responsible for the pathway of care of the chronic disease management program for patients without a family physician, or 2) CR follow-up by the nurses working in the medical clinics where patients are followed up, or 3) CR follow-up by homecare nurses for patients with reduced mobility.

### Phase 4: Deliver a comprehensive CR program

#### Need to adapt and develop nutrition and kinesiology services

In a context where professional human resources are more limited in certain patient localities, participants mentioned strategies to compensate for the absence of specific specialized resources, such as nutrition and kinesiology services. The strategies reported by participants included the importance of collaboration with the tertiary center to offer nutrition and kinesiology services. Participants recommended that these services be provided virtually in synchronous mode. *" Instead, the tertiary centre should help us if there are no resources available to give all patients in the region the same service"* [nurse, site 1]. As far as support from tertiary centers is concerned, all knowledge users were positive that this would be favorable to the CR offer.

Another strategy reported by participants is to consider establishing partnerships with university teaching institutions in programs that train healthcare professionals. This would make up for the shortage of professionals in kinesiology and nutrition, for example, through supervised internships. This recommendation made by the participants would enable a virtual service delivery modality in synchronous mode for kinesiology and nutrition services without overloading tertiary centers. This would allow patients far from healthcare centers to be monitored by students in a university professional program and their supervisors. At the same time, they await these services to be provided nearby. *" It is easier for me to partner with the regional university than to find healthcare professionals"* [Decision maker, site 2].

Participants reported the importance of having technical support available to resolve issues if virtual modalities are implemented. For healthcare professionals working in the tertiary center, training on virtual modality delivery was also cited as an essential element.

Finally, to support patients during CR with healthcare professionals, participants mentioned the importance of patients having a peer or caregiver to help with information retention. *" Patients should have someone accompanying them to help with retention and understanding of information"* [nurse, site 1 and 2].

### Phase 5: Conduct final CR assessment

Professionals reported that the professional judgment of the clinician (e.g. nurse, nutritionist and kinesiologist), according to his or her area of expertise, must always prevail in carrying out the essential components of CR. Participants reported that it does not appear to be the responsibility of managers to decide on the number of encounters established for patient services in phases 4 and 5. Participants reported the importance of assessing patients' acquired skills after the CR program to support shared decision-making to review certain essential CR components in the therapeutic patient education (TPE) process. Based on this assessment, the clinician will always be able to offer additional services to patients with greater support needs. Participants reported the importance of consolidating ununderstood and mastered learning to pursue long-term behavior change. *" We should not record encounters by numbers; instead, we should use our judgment to evaluate the patient's needs "* [nutritionist, nurse, site 1].

### Phase 6: Discharge and transition to long-term management

The participants mentioned that after all phases of CR have been completed, a final report of the data derived from changes in acquired knowledge, anthropometric values and laboratory data from the patient's pre- and post-CR blood tests should remain in the patient's file. However, the final report should also be sent to the physician who referred the patient to CR. The nurse who performed the CR is responsible for completing the final report with the patient's data in phase 5 in collaboration with the other healthcare professionals involved. *"I think the report should be provided by the nurse in charge of cardiac rehabilitation "* [nurse, manager, site 1 and 2].

Participants reported that after completing the CR, primary care services must be the gateway for CVD follow-up for patient needs. Managers reported that primary care services will need to be accessible to patients without a family physician so that they can access healthcare professionals and avoid emergency room visits. *" The gateway after cardiac rehabilitation should be the family physician and primary care nurse"* [manager, site 1].

### Presentation of the final prototype to participants

Participants found the proposed RC prototype acceptable and safe: " *This program reflects our reality as a remote region* " [manager, site 1 and 2]. Nurse practitionner agree with manager " *It's in line with our discussions; I don't see how we could drop patients; it's our context and reality* " [nurse practitioner, site 2]. " *The work [program] is done in the ultimate interest of the patient, and it is he who will benefit from it* " [decision maker, site 2].

Participants felt that the development of the prototype CR program reflected the needs of patients and was consistent with the ability of healthcare professionals to deliver CR in areas far from tertiary centers. Patients reported that the 6-phase prototype program should be implemented quickly. There was a consensus among participants to prioritize rapid management by the nurse and CVD coordinator: " We paid it forward; it's *up to the organization and managers to do the rest* " [patients, site 1]. The nutritionist agrees with the patient.

## Discussion

Concerns and service issues were relatively the same, which enabled us to create, after the cycles, a single prototype for both regions. The patient referral process within a pathway of care was the most important issue to be developed for these two sites to enable the recruitment of all patients navigating through different hospital centers. The results allow us to make the following observations: 1) The service offer for CVD patients in CR for remote regions must differ from the models suggested for specialized centers; 2) It is important to prioritize a varied CR offer for remote regions; and 3) The co-design process enables an inclusive approach in the adherence of all knowledge users to the common principles of CR.

### Service provision for CVD patients in remote areas in CR must differ from suggested models for specialized centers

Health guidelines are essential guides for directing health services. They play a major role in health policy development and healthcare delivery [32].

In this study, we used the guidelines of the CR care model developed by the BACPR [4], as they offer an exciting guide when it comes to developing CR in a territory. Our results emphasized that guidelines are insufficient to drive change in the services provided in remote areas without professional resources. These guidelines need to be tailored to the specific realities of remote areas [4]. A comprehensive and personalized approach is crucial for developing remote cardiac rehabilitation programs [33]. Since living far from specialized centers creates barriers to participating in CR, exploring the provision of CR synchronously using digital technologies is crucial. The literature shows the feasibility of synchronous/real-time digital CR interventions in enhancing the overall cardiac profile of patients [33]. According to the participants in our study, this model is not adapted to the context of remote regions, as patients navigate through different facilities and other regions that offer percutaneous coronary intervention. The fact that patients must navigate through different healthcare facilities complicates the referral and recruitment process. Our study demonstrated the importance of providing a service pathway with a single point of contact who will receive all patient referrals from the different healthcare facilities not to lose patients at discharge. The BACPR recommends a coordinated referral process for CR, and the literature suggests a CR nurse coordinator [34]. In remote areas, a CVD nurse coordinator is implemented in phase 1 to receive all patient referrals for CR from different healthcare facilities. In addition to referral and early recruitment, the nurse performs an early clinical assessment of the patient to identify any health problems since hospital discharge. In the event of health problems since hospitalization, the CVD nurse coordinator can refer the patient to the cardiology clinic for a consultation with a physician, thus avoiding emergency room visits.

Hospital care after a cardiac event and the pathway of care can take up to seven days in remote areas before a patient returns home, compared to 24 to 48 h for a patient in an urban center [10]. According to BACPR guidelines, this makes it difficult to expect care and to start phase 1 of CR in less than ten working days.

One element that emerges from this study is decentralizing CR services from tertiary centers to the patients' community to gain expertise in each locality. This allows greater autonomy for local sectors to initiate changes in services to meet population needs and enable greater proximity of services [35]. The decentralization of CR services to patients' communities makes it possible to personalize patient care and maintain the proximity of care and services [36].

To reach all patients who have relocated away from the tertiary center, our CR program recommends three offers of follow-up by a nurse via nursing services. To achieve this, a nurse would be assigned, depending on the patient's sector of activity and CR registration status, from phase 2 to complete the CR program in phase 6. Firstly, customization of the CR program in phase 2 will have to be carried out in each of the patients' communities, making it possible to better respond to needs and adapt CR for patients with the available human resources. Our results demonstrated that we also need to recognize the cultural issues that are present in each of the patients' communities. Nurses in these communities are more familiar with available community resources and physical activity services. However, they also have access to the medical resources available to the patient for referral when his or her health condition requires medical follow-up. In addition, having three follow-up nursing services will allow us to reach more patients within the region.

### The importance of a varied CR offering for remote regions

The results of this research underline the importance of developing a prototype CR program for remote regions that mobilizes a varied CR offer to democratize CR expertise across the entire healthcare territory. Furthermore, participants reported that there may be services in phase 4 for the basic educational components in person but that telehealth in synchronous mode could also be offered. Offering telehealth in CR is a formula that can help avoid certain service breakdowns [37]. Before using this method, professional training and preparation are required, and interventions should be offered within interdisciplinary perspectives [38]. However, the telehealth CR delivery modality may only suit some patients and geographical areas [38]. We found in our study that healthcare professionals favour telehealth but are not trained to do so and need technical support to ensure that encounters run smoothly. This study also highlights that, although CR is delivered by an interdisciplinary team comprising a nurse, a kinesiologist and a nutritionist, this is more difficult to achieve in low-resource settings.

This is why finding solutions to compensate for the lack of resources and support patient safety is important. A health assessment is necessary to safely transition patients to telehealth to ensure that cardiac rehabilitation (CR) is safe. Additionally, it is important to provide follow-up care to prevent the discontinuation of the CR program and to be able to assess any chest pain during exercise [39].

For example, consider involving primary care nurses who could initiate safe, educational interventions to help patients in their activities of daily living [40, 41]. Nurses working in primary care have the scope and skills to monitor patients for CR [40, 41]. They can perform nursing monitoring of patients after coronary intervention in relation to assessing a symptomatic person's physical and mental condition [40, 41]. They can also support the individual in the self-management of heart disease [42] at every stage of a CR pathway of care [10]. The nurse has the skills to support the patient in navigating the healthcare system and refer him or her, depending on the patient's resources, for private or public services [43].

### The co-design process enables an inclusive approach in which all knowledge users adhere to the common principles of CR

The UCD-based co-design process [30] enabled the development of a prototype for a CR program in remote areas by the knowledge users, who judged that the prototype met their context and needs. This process connected participants' knowledge, skills, and resources to develop a prototype program geographically adapted to the remote environment. The participants could reflect on the different ways of offering a CR service that corresponds as closely as possible to their reality. Participants appreciated being involved in a co-design approach to problem-solving for CR services. The advantages of co-design for the knowledge-users involved were that it fostered the creativity of all the participants, resulting in a prototype model of a CR program that meets their needs, which may facilitate implementation [28]. Involving a number of healthcare professionals from different disciplines and backgrounds gave us an insight into the reality of each setting and the capacity of the services they can offer [28].

### Study strengths and limitations

One of our strengths is that the co-design process took place on two sites simultaneously with similar results on both sites, enabling the transferability of results to similar contexts. In line with previous co-design studies [44, 45], this study offers an example of participatory research but still involves users with different perspectives, including patients, nurses, nutritionists, kinesiologists, managers and directors. One of our study's strengths is that we have developed an RC program within a pathway of care that allows us to take the patient from hospitalization to completion of the RC program. Moreover, we respected remote regions' demographic and professional resource issues so that the RC program would be as responsive as possible for future implementation.

One of our study's strengths is that we have developed an RC program within a pathway of care that allows us to take the patient from hospitalization to completion of the RC program. Moreover, we respected remote regions' demographic and professional resource issues so that the RC program would be as responsive as possible for future implementation. One of the limitations observed in our study is the absence of the decision makers' perspective on the organizational changes that will need to take place in order to know what should be in place to implement this CR program. We would have liked to know the barriers and facilitators when designing this CR program. According to Steen et al. [46], in the development of new public policies, it is important to integrate leaders in co-design in order to bring about a cultural and structural change in practice for the application of new knowledge. The focus was on healthcare professionals and patients, who said they wanted the program to be implemented quickly. However, there was a lack of participation at the organizational level by decision-makers and managers, which is fundamental to bringing about changes in practice. Thus, we were unable to collect data on organizational change strategies for the future implementation of this program.

## Conclusion

This study describes the development process of a CR program for two remote regions. We described the use of a user-centered co-design process to adapt knowledge to local contexts. The CR program developed in partnership with knowledge users, including patients, could support the provision of CR services in remote areas. One of our study's strengths is that we have developed an RC program within a pathway of care that allows us to take the patient from hospitalization to completion of the RC program. Moreover, we respected remote regions' demographic and professional resource issues so that the RC program would be as responsive as possible for future implementation. Subsequent researches are needed to develop a conceptual framework for collaboration between tertiary cardiology centers and primary care in remote areas.

## Data Availability

The data that support the findings of this study are available from CIUSSS du Saguenay-Lac-Saint-Jean research center, but restrictions apply to the availability of these data, which were used under licence for the current study and so are not publicly available. The data are, however, available from the authors upon reasonable request and with the permission of the research team.

## Abbreviations

BACPR: British Association for Cardiovascular Prevention and Rehabilitation
CR: Cardiac rehabilitation
CVD: Cardiovascular disease
HAS: Hautes Autorité de santé
PCI: Percutaneous Coronary Intervention
UCD: User-centered design
TPE: Therapeutic patient educational
